# Supplementary material for: A fuzzy description logic based IoT framework: Formal verification and end user programming
Source: PLoS One. 2024 Mar 22;19(3):e0296655. doi: 10.1371/journal.pone.0296655 (PMC10959344; doi:10.1371/journal.pone.0296655)
Supplement: Attachment — Submitted filename: Review Comments_Aug23.docx [file pone.0296655.s001.docx]

**Review Comments**

Thank you for the opportunity to peer-review this paper. This paper proposes a structure that allows non-technical users build IoT applications to meet their needs. The structure uses Fuzzy Logic reasoner which transforms human language into executable action to actuators. The structure/framework has the benefit of being user-friendly and easily understood. It can be operated by people with no prior knowledge and has measures in place that can prevent inconsistencies within the system which will in turn make the system consistent and reliable.

The following observations have been raised;

1. In the abstract (line 11), the word built should be changed to build (to build their own IoT applications).
2. The headings and subheadings are not numbered/lettered.
3. The paragraph (lines 46-53) has no citation. Also, lines 54-59. Cite appropriately.
4. Fig 2, Fig 4, and Fig 5 are blurry on some parts. Clearer images should be uploaded with better resolution.
5. In the section of Actuator system (line 377), the statement is ambiguous. Kindly paraphrase.
6. The performance and efficiency of the fuzzy logic reasoner blocks in real-world applications have not been outlined in the paper.
7. Only a smart-home IoT security system was considered in the paper. As such, it is uncertain how the framework would function when used for other applications.
8. There is no performance evaluation of the framework. Having this will enhance the quality of the paper.
